# When the going gets rough – studying the effect of surface roughness on the adhesive abilities of tree frogs

**DOI:** 10.3762/bjnano.7.201

**Published:** 2016-12-30

**Authors:** Niall Crawford, Thomas Endlein, Jonathan T Pham, Mathis Riehle, W Jon P Barnes

**Affiliations:** 1 Centre for Cell Engineering, Institute of Molecular Cell and Systems Biology, University of Glasgow, Glasgow, Scotland, UK; 2 Max Planck Institute for Intelligent Systems, Stuttgart, Germany; 3 Max Planck Institute for Polymer Research, Mainz, Germany

**Keywords:** adhesion, friction, *Litoria caerulea*, roughness, tree frog

## Abstract

Tree frogs need to adhere to surfaces of various roughnesses in their natural habitats; these include bark, leaves and rocks. Rough surfaces can alter the effectiveness of their toe pads, due to factors such as a change of real contact area and abrasion of the pad epithelium. Here, we tested the effect of surface roughness on the attachment abilities of the tree frog *Litoria caerulea*. This was done by testing shear and adhesive forces on artificial surfaces with controlled roughness, both on single toe pads and whole animal scales. It was shown that frogs can stick 2–3 times better on small scale roughnesses (3–6 µm asperities), producing higher adhesive and frictional forces, but relatively poorly on the larger scale roughnesses tested (58.5–562.5 µm asperities). Our experiments suggested that, on such surfaces, the pads secrete insufficient fluid to fill the space under the pad, leaving air pockets that would significantly reduce the Laplace pressure component of capillarity. Therefore, we measured how well the adhesive toe pad would conform to spherical asperities of known sizes using interference reflection microscopy. Based on experiments where the conformation of the pad to individual asperities was examined microscopically, our calculations indicate that the pad epithelium has a low elastic modulus, making it highly deformable.

## Introduction

Tree frogs exhibit excellent climbing abilities which allow them to efficiently move through their typically arboreal habitat, doing so using specialised adhesive pads found distally on the ventral surface of each toe. The pads stick by means of ‘wet adhesion’, whereby a thin fluid layer is produced by the pad which creates capillary and viscosity forces between the pad and the surface [1–3]. The polygonal epithelial cells (approx. 10 µm in diameter) are covered with nanostructures, which are thought to create friction by direct contact with the surface [3]. The combination of the fluid filled adhesive area and the specialised morphology allow the tree frogs to climb smooth vertical and overhanging surfaces.

The attachment ability of tree frogs is affected by both surface chemistry and surface roughness. Hydrophobic leaves (such as those on lotus leaves [4]), could affect the capillary forces produced by the pad (which require low fluid contact angles), and deprive the pads of adhesive ability. Indeed, many plant surfaces are hydrophobic, as this reduces water loss [5]. Turning to surface roughness, this affects the ability of the pad surface to form close contact with the surface. With fine-scale roughness, the volume of fluid produced by the toe pads may be sufficient to completely fill the gaps between the asperities (the ‘flooded‘ regime described by Bhushan [6]), in which case adhesion will remain good. However, with larger asperities, this is no longer possible (‘toe-dipping‘ and ‘pill-box‘ regimes), and capillary forces will be reduced due to a decline in the Laplace pressure component of capillarity [7]. Bhushan’s ‘submerged‘ regime is that of a rock/torrent frog climbing water-covered rock, where the toe pads are completely submerged, thus abolishing any meniscus [8]. In such cases, capillary forces will be absent, and any adhesion will be likely due to rate-dependent viscous forces.

Roughness is a component of surface texture, a measure of the amplitude and frequency of deviations from a flat surface. Most natural surfaces are not smooth (unless polished by, for instance, the action of water), but will have a roughness that reflects the size of the component particles (e.g., sand grains in sandstone) [7]. They may also contain cracks, lumps and ridges which increase their roughness still further. Leaf surfaces may be relatively smooth, but their veins give high amplitude ridges that are distributed over their surfaces. Indeed, cuticular folds have been demonstrated to be slippery for beetles [9–10], and stomata also contribute to a leaf’s roughness. Additionally, on some plants (e.g., the stems of *Macaranga* trees), one may find epicuticular wax crystals [11]. In *Macaranga*, the resulting slipperiness repels all insects except their specific ant partners [12]. Rough surfaces could also be abrasive and therefore potentially damaging to adhesive surfaces.

Despite extensive research on the adhesive abilities of tree frogs, most studies have involved testing their climbing capabilities on smooth surfaces [2,13–14]. On such surfaces, the toe pad fluid creates an ultra-thin layer, whilst the nanopillars that cover the surface of the pad epithelial cells come into very close (potentially direct) contact with the surface [3]. Thus the presence of surface asperities is likely to have a significant effect on adhesive ability. Early work by Barnes and co-workers [15] showed that tree frogs display minimum adhesive ability on an intermediate roughness which was larger than their cell morphology but smaller than the pad itself. Torrent frogs can stick well to wet and rough surfaces similar to their waterfall habitats, but both torrent frogs and tree frogs showed a decrease in performance as asperity size increased under dry conditions [8]. Other studies, including that of Emerson and Diehl [16], studied sticking on surfaces that varied in both roughness and chemistry, making precise conclusions difficult.

The pads of tree frogs are very soft and so should deform to mould around rough surfaces, as is seen in smooth padded insects [17]. The Young’s modulus of the toe pads has been measured in several studies, an elastic modulus of 40–55 kPa based on AFM indentation being the most recent estimate [18]. Barnes et al. [19] carried out indentations at different depths and measured different degrees of stiffness at different depths, lower values for the elastic modulus resulting from larger indentations. This is probably due to the stiff outer keratinous surface of the pad. The toes also have extensive blood vessels beneath the pads which will contribute to the soft nature of the whole pad [19]. It is, however, unknown to what extent these soft pads can deform and adapt to different scales of roughness.

Here, the performance of tree frogs on rough surfaces was examined using a variety of techniques to test sticking ability, both at the toe pad level and in free climbing tree frogs, using different rough surfaces. In the main, polishing discs and sandpaper were used, since the range of roughnesses found in such materials was closest to the natural surfaces that a climbing tree frog would encounter. For the single toe pad force measurements, both translucent resin replicas of the sand and polishing papers and fabricated surfaces of known dimensions were used. The hypothesis tested was that, on rough surfaces with larger asperities (i.e., larger than the pad cell diameter of 10 µm), the increased cavity area between the pad and surfaces would be such that pad fluid secretion would be unable to fully fill the pad contact area, as proposed by Persson [20]. Such a situation would be reflected in a reduction in adhesive force. To gain further insight into this, interference reflection microscopy (IRM) was used to view the pad surface and observe how well it conformed to single asperities (glass beads) of different sizes by measuring the height profile of the pad conforming around particles, and observing at what asperity size air bubbles initially appeared.

## Results

### Attachment abilities of free climbing tree frogs

**Figure 1:**
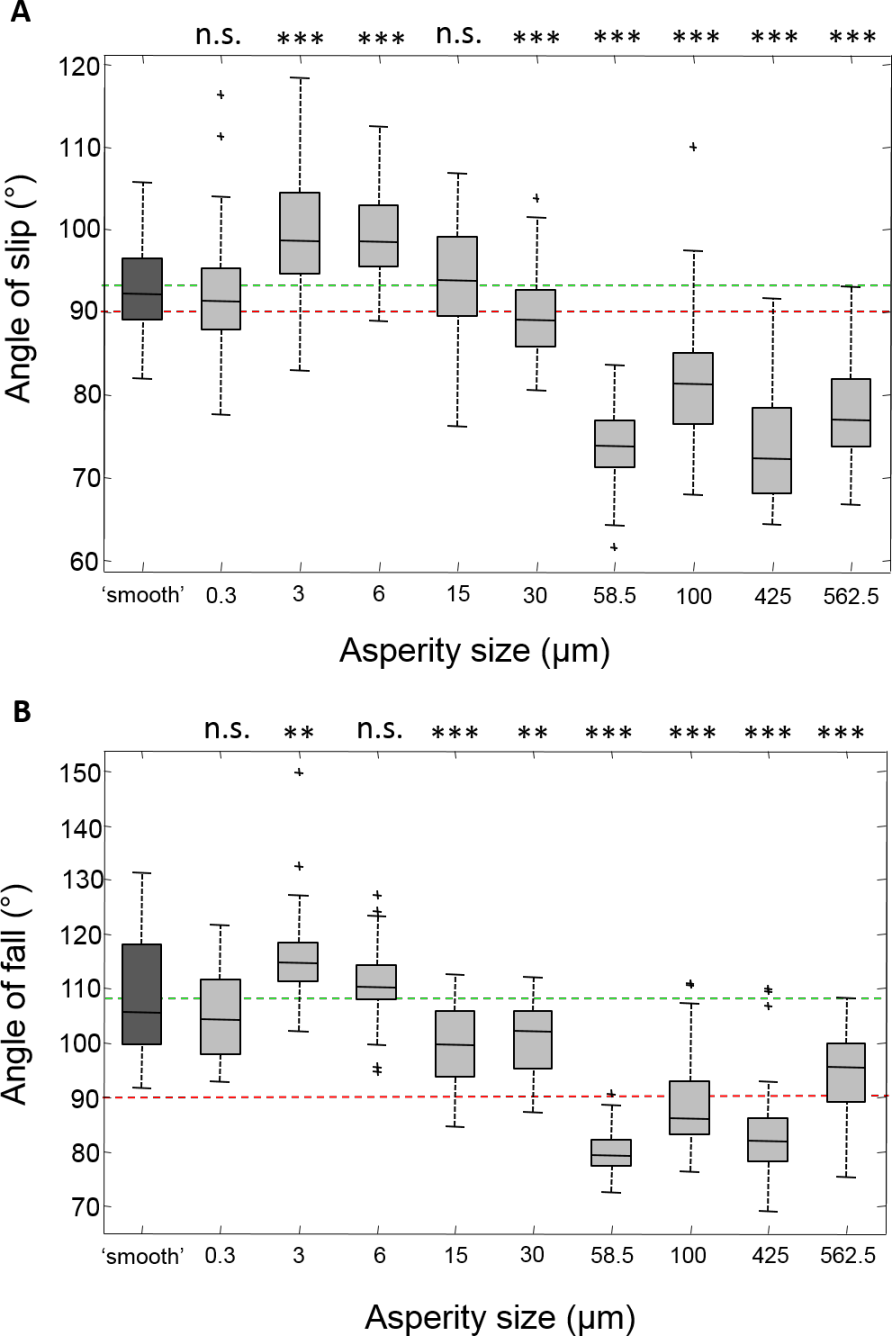
Box plots of slip (A) and fall (B) angles of free climbing tree frogs on different rough surfaces. Smooth glass is on the left, with increasing roughness (larger asperities) moving right across the *x*-axis. The red dashed line through 90° on the plot shows where friction forces are at a maximum, and where adhesive forces begin to play a role. The green dashed lines show mean values on the smooth surface to aid comparisons. Statistical tests which compare each surface with the smooth surface performance are shown above each box (due to the Bonferroni correction: 95% confidence interval *p* = 0.0055 (*), 99% *p* = 0.0011 (**), 99.9% *p* = 0.00011 (***), n.s. = not significant).

The frogs’ climbing abilities were tested on different rough surfaces using the tilting board apparatus described in the Experimental section (*n* = 60 for all surfaces tested). At the beginning of the test, frogs usually exhibited a relaxed and crouched posture with all legs tucked under the body. However, as the angle of the board increased, frogs would typically spread their limbs in order to help stay attached. This behaviour (which has been described in previous studies and is not unique to this experiment) helps in producing friction forces whilst keeping the peel angle of the pads low [21]. A ‘smooth’ glass surface was used as a control for the rough surface tests. Boxplots are shown below, comparing the performances of the frogs frictional (Figure 1A) and adhesive forces (Figure 1B). Mann–Whitney *U* tests were conducted on comparative sets of data (*n* = 60), with a Bonferroni correction implemented for multiple data usage.

Slipping behaviour, an indication that frictional forces have reached their maximum, was generally not seen on the smooth surface until after 90° had been reached, and occurred at 92.89 ± 5.05°. All tests were compared to the smooth surface performance, which was the control surface. The frogs performed best on the smaller scale roughnesses, not slipping until a higher angle of 99.5 ± 7.44° on the 3 µm; this is significantly higher than the performance on the smooth (*z* = −4.9915, *p* < 0.0001). A similar result was seen on the 6 µm surface (*z* = −5.7368, *p* < 0.0001). As the roughness of the surfaces increased, this resulted in a decrease in the angle of slip. Slipping occurred before vertical (mean of 89.4°) on the 30 µm surface, significantly lower than on the smooth surface (*z* = 3.6554, *p* < 0.0001). On the largest roughnesses, frogs performed poorly, with the frogs failing to produce much friction and slipping at comparatively low angles.

The angles at which the frog fell off the surface are a measure of the maximum adhesive force produced by the frog (Figure 1B). As with friction, the frogs performed well on the smaller scale roughness, but poorly on the rougher surfaces. On the smooth surface, the frogs fell from the platform at 108.7 ± 10.9°, staying attached beyond vertical where the surface becomes an overhang. The best adhesion occurred on the 3 µm surface, the frogs staying attached until 115.2 ± 7.2° (*z* = −3.388, *p* < 0.0007). On the larger scale roughnesses (58.5 µm, 100 µm and 425 µm), the frogs usually failed to reach 90° and therefore seldom tested their adhesive ability. For the roughest surface (562.5 µm), there appeared to be some recovery, with frogs managing to stay attached until 94.9 ± 7.5° and showing some adhesive ability.

To summarise the tilting experiment, the tree frogs show significantly better performance on the smaller scale roughness (3–6 µm) compared to the smooth glass surface. However, on larger roughnesses (58.5–562.5 µm) the frogs performed worse, with frogs slipping and falling at significantly lower angles than on the glass.

### Individual toe pad force measurements

**Figure 2:**
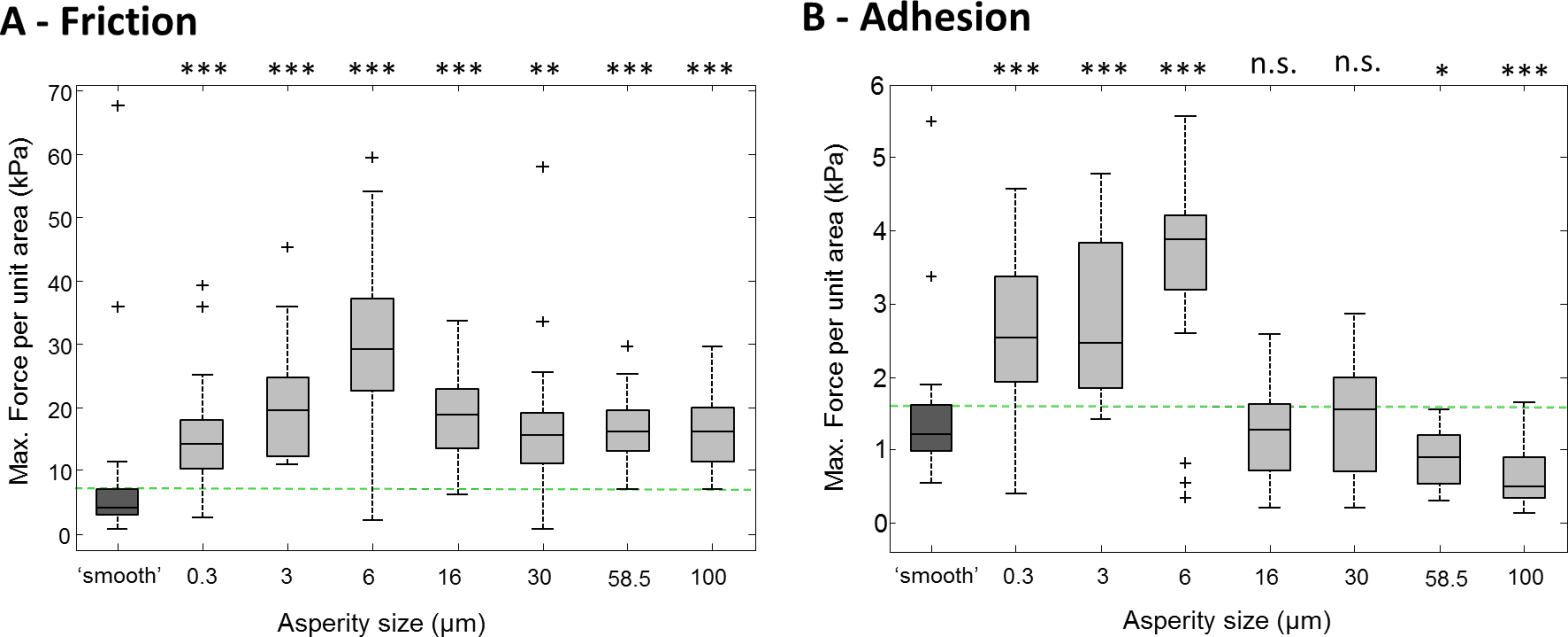
Single toe pad forces on rough surface replicates. Force per unit area has been calculated for friction (graph A) and for adhesion (graph B). Performances on surfaces of varying roughness (approximate asperity heights) are compared with ‘smooth’ surface performance (dark grey box, left). The green dashed lines show mean values on the smooth surface to aid comparisons. Statistical tests are denoted above each box (due to the Bonferroni correction: 95% confidence interval *p* = 0.0071 (*), 99% *p* = 0.0014 (**), 99.9% *p* = 0.00014 (***), and n.s. = not significant).

In order to understand the performance of unrestrained frogs described above, the friction and adhesion of individual toe pads was measured under controlled conditions where contact area was recorded and defined surface geometries were used (see Experimental section). Single toe pads were tested on different rough surfaces (*n* = 30 for each surface tested), the extracted force per unit area measurements for adhesion and friction being plotted in Figure 2.

On smooth resin surfaces, the pads produced a mean maximum of 7.76 ± 12.9 kPa of frictional shear stress (Figure 2A). Forces initially increased with roughness, with the largest shear stresses being measured on the 6 µm surface (30.1 ± 13.8 kPa; *z* = −5.1672, *p* < 0.00014). Shear stress values on the 15 µm surfaces were 18.48 ± 6.1 kPa, higher than the smooth values (*z* = −5.5663, *p* < 0.00014), but lower than the forces on the 6 µm surface. The shear stress measured on the largest roughnesses tested were at a consistent level of ca. 16 kPa, still higher than those measured on the smooth surface (e.g., comparing smooth to 100 µm, *z* = −5.5072, *p* < 0.00014).

Adhesive forces (Figure 2B) measured were much lower than the friction forces. On the smooth surface they were measured as 1.74 ± 1.9 kPa, with peak adhesive forces occurring on the 6 µm surface (3.72 ± 1.5 kPa), significantly higher than on the smooth surface (*z* = −4.4871, *p* < 0.00014). On the two largest roughnesses tested on (58.5 and 100 µm), the adhesive forces were significantly lower than on the smooth surface, forces of 0.9 ± 0.8 kPa (*z* = 3.0382, *p* = 0.0024) and 0.66 ± 0.6 kPa (*z* = 4.7828, *p* < 0.00014) being measured for the 58.5 µm and 100 µm surfaces, respectively.

**Figure 3:**
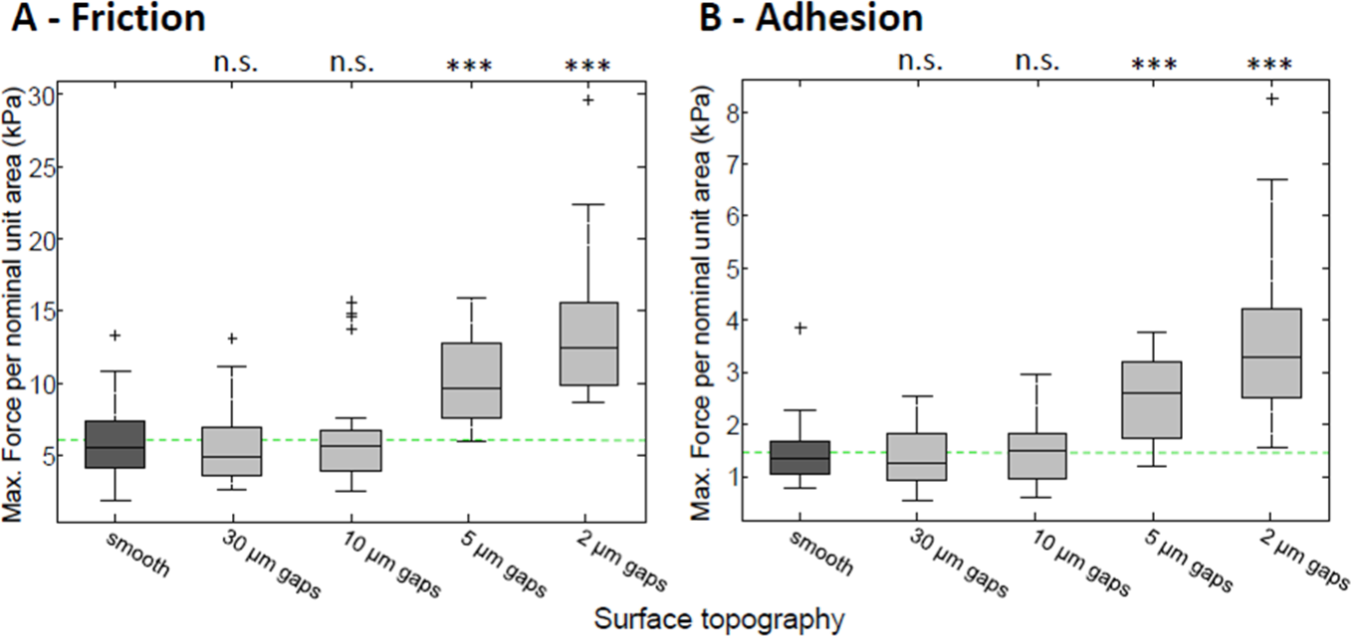
Force measurements of single toe pads on PDMS rough surfaces. The pads force per (nominal) unit area is shown for friction (A) and for adhesion (B), on rough surfaces (varying in the size of the gap between the asperities). They are compared to the forces produced on a smooth surface (dark grey). The green dashed lines show mean values on the smooth surface to aid comparisons. Statistical tests are denoted above each box (due to the Bonferroni correction: 95% confidence interval *p* = 0.0125 (*), 99% *p* = 0.0025 (**), 99.9% *p* = 0.00025 (***), and n.s. = not significant).

Using the same movements on the force plate, a different variation of rough surfaces made from PDMS were tested, where the only parameter changed between surfaces is the gap between the asperities on the surface. Force per unit area measurements on these rough surfaces were compared to forces measured on a smooth PDMS surface (Figure 3).

Shear stress values (*n* = 30) measured on a smooth PDMS surface (5.94 ± 2.6 kPa) were similar to those measured on the resin smooth surfaces. The highest friction forces were measured on the 2 µm gapped surface (13.7 ± 4.9 kPa), significantly higher than the smooth surface forces (*t* = −7.6879, *p* < 0.00025). An increase in the gap size resulted in shear stress gradually returning the levels seen on a smooth surface. Adhesive forces on the PDMS surfaces (*n* = 30) followed the same pattern as the shear stresses, with a peak of adhesive forces seen on the 2 µm gapped surface. Forces reached 3.49 ± 1.5 kPa, which was higher than the smooth values of 1.43 ± 0.6 kPa (*p* < 0.00025). An increase in the gap between pillars resulted in adhesive stress returning to smooth surface values.

To sum up the force measurements on individual toe pads, frictional forces are consistently higher than adhesive forces (>10 times so in some cases). The adhesive and frictional forces of the pads do, however, behave similarly on the rough surfaces. In particular, on the resin replicas, they both increase when roughness occurs on the small scale (i.e., on the 3 and 6 µm surfaces). At higher levels of roughness, shear stress, although lower, plateaus at a level above that seen on the smooth surface, while adhesive force declines to a lower level than on the smooth surface. Although the height of all pillars on the PDMS surfaces is the same, the gaps between them are varied. Surfaces where the pillars are close together are, in relation to the toe pads, rougher than when pillars are widely separated. Thus the highest forces are seen when the pillars are close together, the force values declining as the gaps between pillars are increased.

### Using IRM to visualise pad contact

**Figure 4:**
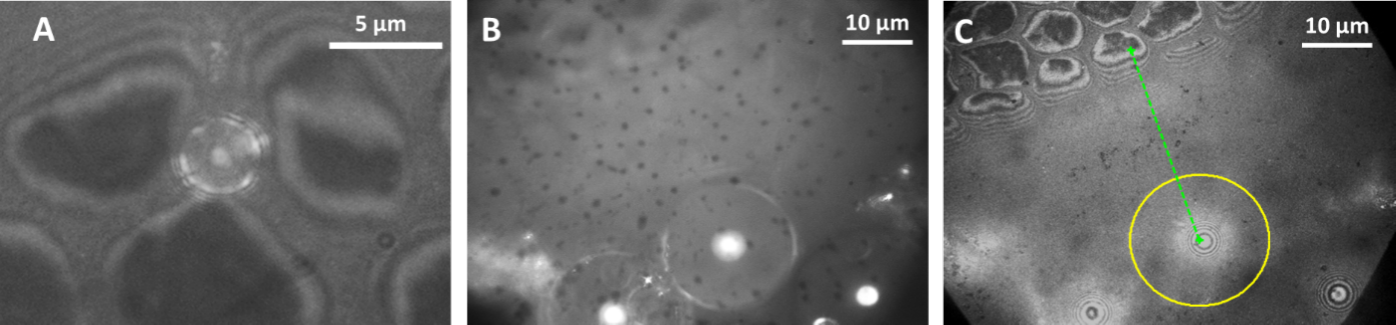
IRM images of the toe pad in contact around a glass bead. Interference fringes indicate the pad sloping away from the glass surface, due to the presence of a bead nearby. A) displays how smaller beads get trapped in the channels between the cells of the pad. B) and C) show two images taken at different focal planes; B) allows the circumference of the bead to be seen, which can then be recorded and superimposed on the second focal plane (C). The horizontal distance from the centre of the bead to the centre of the closest cell in close contact with the glass is marked by a green dashed line. The dark central part of each toe pad epithelial cell is the zero order dark fringe, representing a pad-glass distance of at most a few nanometres and is used as the starting point of the distance measurement, while the centre of the bead is the end point.

**Figure 5:**
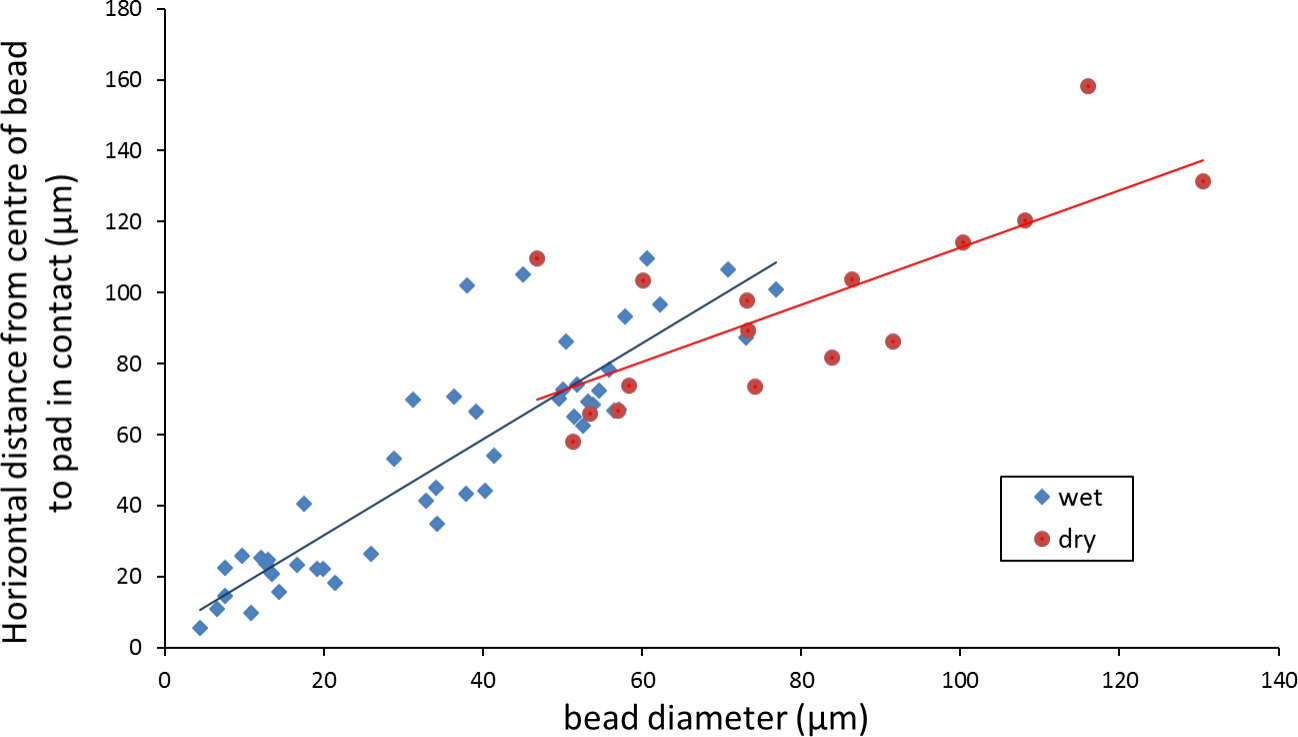
Scatterplot showing correlation between asperity size and pad contact (distance from bead centre to pad in contact with the surface). Data are differently coloured depending on whether fluid completely filled (‘wet’) or only partially filled (‘dry’) the gap created between the pad, bead and surface. In the latter case, the remaining space was filled by an air bubble.

Using IRM (see Experimental section) allowed the pad/substrate contact to be visualised, where the polygonal cells of the pad can be seen to be in close contact with the surface, with channels between the cells to allow the flow of pad fluid throughout the contact area (Figure 4). This corresponds with toe pad studies previously conducted by Federle et al. [3], and allows pad/surface distances to be estimated at a cellular level. For this experiment, glass beads of different sizes were used as asperities on the surface, and the extent to which they disrupted the normal close contact of the pad to the surface recorded. The horizontal distances between the centre of each asperity and the nearest epithelial cell in close contact with the surface were measured and are plotted in Figure 5. The data has been labelled depending on whether fluid completely filled the gap between the pad, asperity and glass surface (‘wet’), or whether an air pocket was present (‘dry’).

**Figure 6:**
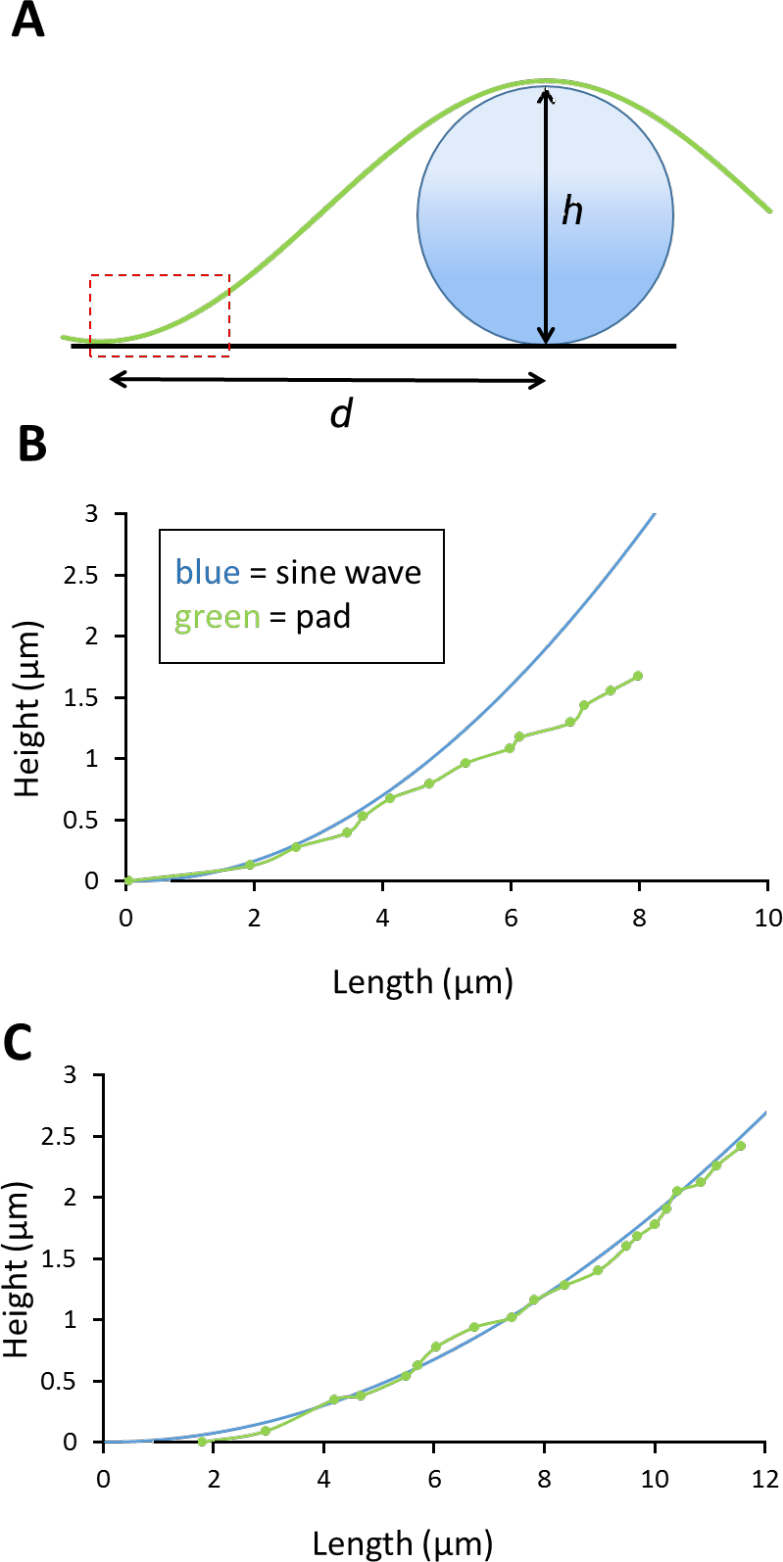
Modelling of the contact behaviour of the pad around a spherical asperity. A) Conceptual drawing showing the parameters used for the model. A sine wave with wavelength 2*d* was used to fit a spherical bead with diameter *h*. The initial contact of the pad separating quickly from the surface can be seen using IRM (indicated by the dashed box). B) for smaller bead sizes (here *h* = 32.79 µm) the data points only follow the sine wave initially (before showing a slope underneath the sine wave curve, indicating a soft material) whereas the fit is better for larger particle in example C (*h* = 57.92 µm).

A general linear trend can be seen in the entire data, showing that, as bead size increases, the gap between the bead and the nearest point of pad close contact with the surface increases too (Spearman correlation test; *Rho* = 0.7307, *n* = 64, *p* < 0.001). For smaller bead sizes of up to 50–75 µm, the pad fluid completely fills this gap, but for larger beads, this is no longer the case and air bubbles are seen. This indicates that the size of the asperity affects whether fluid can fill the gap created by the asperity (comparison of gap distances from ‘wet’ and ‘dry’ gaps: Mann–Whitney *U* test, *n* = 48, *z* = 3.0466, *p* < 0.001). Interestingly, correlation tests on the separated data – fluid filled (‘wet’) or air bubbles present (‘dry’) show a significant linear correlation for the ‘wet’ measurements (Spearman correlation test; *Rho* = 0.7866, *n* = 48, *p* < 0.001), but not for the data points where fluid didn’t fully fill the gaps (Spearman correlation test; *Rho* = 0.3947, *n* = 16, *p* = 0.1303). The fringes seen at the edge of the pads allow the initial slope of the pad (a linear fit through the first 4 data points, starting from the pad) as it leaves the surface to go over the bead to be measured. For all beads tested, a similar slope of the pad was seen (mean slope = 0.21 ± 0.09; *n* = 64), which indicates that the pad is a highly soft material that can mould to the surface consistently. Indeed, the similarity of some of the measurements (Figure 6) to a sine wave indicates that bending occurs gradually (i.e., without any kinks). The degree of bending reflects the elastic modulus of the toe pad epithelium, an estimate of this appearing in the Discussion section.

### Climbing on a rough and wet surface

**Figure 7:**
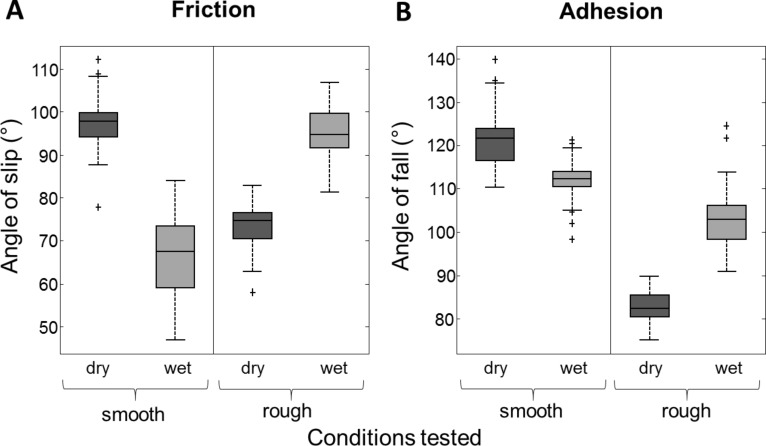
Boxplots displaying attachment performance of tree frogs in varying conditions. The angles of slip (A) and fall (B) of the frogs are shown for dry and wet surfaces, which could also be either rough or smooth.

Our experiments involving IRM indicated that frogs may be unable to produce sufficient pad fluid to fill gaps between asperities on the roughest surfaces. This would mean a loss of attachment ability as occurred on the rougher surfaces in the tilting experiment. Therefore, an additional test was done to see whether the addition of fluid to a rough surface increases the frictional and adhesive abilities of tree frogs (4 frogs used for experiments, *n* = 40). This was done using the tilting board apparatus, comparing the performances of frogs on a smooth glass surface and on a rough sandpaper surface under both dry and wet conditions. The 58.5 µm surface was used, as the frogs had performed poorly on this surface in the previous tilting experiment; it was also around this asperity size that air bubbles began to appear in the IRM experiments. For the wet conditions, water was sprayed onto the surface (using a water spraying mister) prior to each run of the test. As before, the angles at which the frogs slipped and fell were recorded, which relate to their frictional and adhesive abilities, respectively. Results for this experiment are shown in Figure 7.

The angles of slip and fall on the dry smooth surface (97.6 ± 6.2° for slip and 121.2 ± 6.1° for fall) were broadly similar to the results described previously (in the free climbing tree frog section). Likewise, the frogs also attached poorly to the dry rough surfaces (slip angle: 73.4 ± 4.98°, fall angle: 82.8 ± 3.7°). However, when water was introduced to the smooth surface, it caused a loss of friction in the frogs’ pads. This lead to the frogs sliding at relatively low angles (66.1 ± 9.2°), which is worse than on the dry surface (*z* = 7.6739, *p* < 0.001). In contrast, when extra fluid was added to the rough surface, the frictional performance significantly improved from when the same surface was dry (Student’s *t*-test: *t* = −18.3666, *p* < 0.001), so much so that the slip angle performance on the rough wet surface (95.6 ± 5.8°) did not significantly differ from the slip angles on a dry smooth surface (*z* = 1.5348, *p* = 0.1248).

For the angles of fall (representing maximum adhesive performance), the performance on a smooth, wet surface was lower than that on a smooth dry surface. However, even though the pads were continually slipping due to low friction, the frogs were able to stay attached until 111.9 ± 4.6°, demonstrating that they retain some adhesive ability. On the wetted rough surfaces, however, the frogs were able to adhere more strongly than when the same surface was dry. They stayed attached until 103.1 ± 7.1° when it was wet, compared to 82.8 ± 3.7° when the surface was dry (*t* = 16.198, *p* < 0.001).

These experiments show that the poor performance of frogs on the roughest surfaces can be significantly improved when the surface is wetted. However, on a smooth surface the presence of water leads to a drop in their climbing abilities, particularly frictional forces.

## Discussion

### Tree frog adhesion

Most evidence supports the hypothesis that tree frogs adhere by capillary forces [2,13,22], but roles for other adhesive mechanisms (such as hydrodynamic forces) cannot be excluded. Indeed, it is very likely that viscosity-dependant hydrodynamic forces do play a role, as torrent/rock frogs that have toe pads like those of tree frogs [23] can adhere to rough surfaces with their toe pads completely covered in running water [8], a situation where capillary forces would be absent. Additionally, since toe pads make close contact to surfaces, a role for van der Waals forces cannot be excluded [3]. However, little evidence was found for such forces in a recent AFM study of the toe pads of *Litoria* [24]. Capillary forces are highest when the volume of fluid is at a minimum, particularly at the air–water interface around the edge of the pad, for it is the curvature of the meniscus that provides the adhesive force, either directly through tensile forces that depend on length (circumference of pad) or on pressure forces (Laplace pressure) that depend on the area under the pad [25].

Since tree frog adhesion depends upon a fluid joint and fluids tend to act as lubricants, it is surprising that tree frogs can generate high friction forces. Such forces are thought to be due to close contact between the tips of the nanopillars that cover the pad surface and the substrate. Indeed, friction forces are much larger than would be predicted by any system involving a continuous fluid layer under the pad [3].

Another important feature of toe pad fluid is its chemical composition. Although we have treated it as water in all these experiments, tree frogs appear to be able to adhere to hydrophobic surfaces just as easily as hydrophilic ones [26], which would not be the case if toe pad fluid were pure water. A preliminary biochemical analysis of the toe pad fluid [26] suggests the presence of carboxylic acids which could act as surfactants, lowering the contact angle, and thus allowing frogs to adhere to even strongly hydrophobic surfaces.

### Our experiments

The whole animal tilting experiments provide direct data about the tree frog’s capabilities on rough surfaces, as the slip and fall angles reflect friction and adhesive forces of the frogs [16]. With slip angles, an angle of 90° represents the maximum friction force that this technique can measure. One might therefore predict that, if a frog did not slip by 90° then it should not slip at all, but simply fall from the platform when the angle for maximum adhesion was reached. This occurred in some cases, but most of the frogs slipped before they fell. The most likely explanation of this is that, at these high angles when the frog’s mass is pulling the animal away from the platform, there is a decrease in actual toe pad contact area. This means that, even if the shear stress continues to increase, the total force will eventually decline and the frog will slip. Adhesion measurements are also limited were the frog to be able to remain attached at 180° (upside-down). This often occurs with small frogs (mass: <5 g) [2], but not with the frogs used in this study.

The force measurements on single toe pads, on the other hand, provide data on the forces that can be generated by the toe pad epithelium and how they are affected by surface roughness. Proximally-directed horizontal pulls provide information on the frictional capabilities of toe pads, while vertical pull-offs give information on adhesion. It is important to remember, however, that what is important to a climbing frog is the pull-off force, which has components of both adhesion and friction for all angles below 90° (and above 0°). The maximum adhesive capabilities of frogs can depend hugely on friction, for friction forces keep the pad/ground angle low, maximising the resultant (pull-off) force and preventing peeling of the pad from the surface [21]. Similar interactions occur in geckos [27]. This means that it is not possible to separate adhesion and friction unambiguously from whole animal tilting experiments, but the data remain useful in showing how surface roughness affects a tree frog’s climbing performance.

As the frogs‘ sticking ability is reliant on wet adhesive forces, the fluid layer beneath the pad in contact with the surface is key to how effectively tree frogs can climb. Interference reflection microscopy (IRM), the third main technique utilised in this study, produces patterns of interference fringes between light reflected from the toe pad/fluid interface and light reflected from the coverslip/fluid interface. The fluid layer thickness can be calculated by comparing pairs of images of the same area of pad using two different wavelengths of monochromatic light [28]. This technique was first used on tree frogs to estimate the thickness of the fluid layer under the pad [3], and used here to study changes resulting from the presence of single asperities (glass spheres of various diameters). Small spheres can fit into the gaps between neighbouring epithelial cells, and air bubbles were present surrounding large spheres. Additionally, measurements of the thickness of the fluid layer immediately surrounding glass spheres of known diameter allowed for estimates of the reduced elastic modulus of the toe pad epithelium to be made (see below).

### Rough surface effects on adhesion and friction

**Figure 8:**
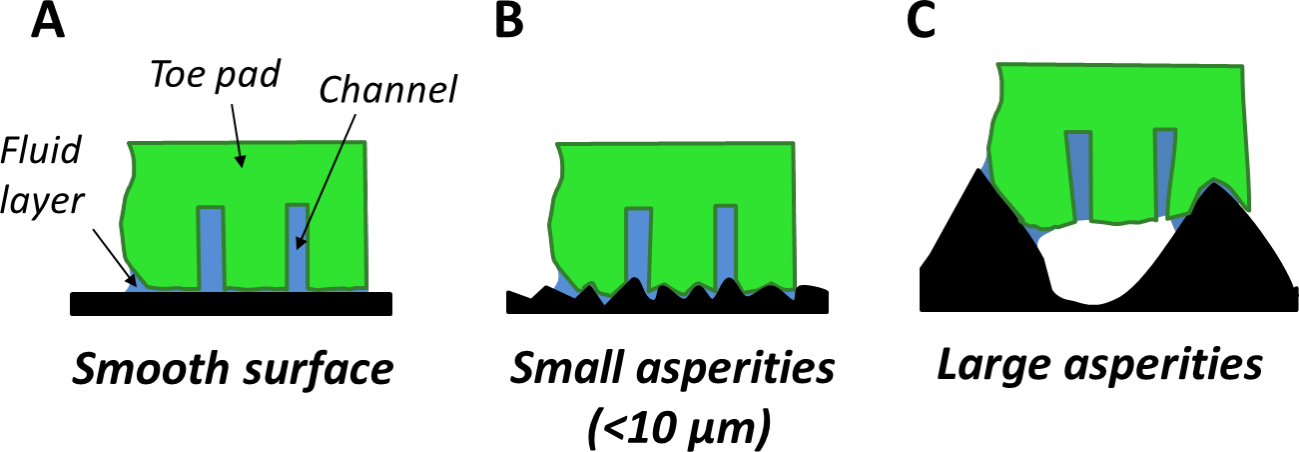
Simplified diagram predicting toe pad contact on different rough surfaces. Contact on a smooth surface (A) is shown, along with the pads conformity to rough surfaces with small (B) and large asperities (C).

**Friction:** Both slip angles and friction forces on the surfaces increased as the roughness increased from very low values (a ‘smooth’ glass plate) (Figure 8A), reaching a peak for wavelengths in the range of 6–12 µm (spacing of asperities is approximately twice their height, as shown below in Table 1). This is in the range of the diameter of the toe pad epithelial cells (approx. 10 µm) and suggests that the large friction increase could have been due to interlocking of the tips of the asperities with the narrow channels that separate the epithelial cells (Figure 8B). Evidence for this comes from the IRM experiments, where the smallest beads used (<3 µm diameter) were often seen within the channels which separate the cells (Figure 4A). On the PDMS surfaces where the only variable is the spacing of the pillars, the increase in friction force was related to the density of asperities, and thus could have been caused by interlocking of the 2 µm diameter asperities with the 1–2 µm channels between the toe pad epithelial cells. An alternative explanation for this increase in friction relates to the fact that the toe pad epithelium can be thought of as a viscoelastic material and, as such, will dissipate energy when it is deformed [29]. Such energy would contribute to the friction force on rough surfaces. Indeed, such viscoelastic deformations can also enhance adhesion [30]. For larger roughnesses on the polishing disc surfaces (particle size ≥ 30 µm), slip angles are well below the values obtained on the ‘smooth’ surface, while single toe pad friction forces on the replicas remained higher than ‘smooth’ surface forces. Since friction in tree frogs has been shown experimentally to scale with area [31], and real contact area is increased on a rough surface, the increase in the friction force in relation to the glass surface would be predicted. But why did it not occur in whole animal experiments? A clue to the answer comes from the whole animal experiments where wet and dry surfaces were compared (Figure 7). Wetting the surface significantly increased slip angles on rough surfaces, presumably because the added water reduced the occurrence of air bubbles, such as those seen around large asperities in the IRM experiments. In the presence of such air bubbles, contact between pad and surface would be reduced rather than increased (Figure 8C). However, this explanation poses the question as to why air bubbles appear in whole animal experiments but not in the single pad force experiments. It could be explained by tree frogs toe pads not producing much fluid, and leaving some behind after every step. It is likely that a frog trying to hang on to a rotating platform will have a significantly thinner layer of fluid than in the force plate experiments, where only single measurements were made from any one toe pad (see Experimental section).

The tilting experiments of Endlein et al. [8] revealed a similar result, with the increase in roughness leading to lower fall angles under dry conditions. The addition of water led to an improved adhesive performance, but slipping occurred more frequently. Often, these slips were transient, the frogs reattaching after a slide of at most a few centimetres [8]. During such slides, fluid is removed from under the pad, the reattachment potentially occurring when the fluid thickness is sufficiently reduced for close contact to re-occur. As discussed by Hutt and Persson [32], any tendency for the pads to detach will be resisted by the reduction in pressure that develops in the thin film under the pad, which presses the pad to the substrate and therefore acts as a strong hydrodynamic adhesive (viscous adhesion). There must therefore be a fine balance for frogs with respect to the volume of pad fluid that they produce for effective climbing on a wide variety of surfaces. It is unclear how the fluid production is controlled in frogs, but as fluid is often left behind in steps [33], it must be replenished frequently.

**Adhesion:** For both fall angles and single pad adhesive forces, the pattern shows many similarities with slip angle/single pad friction force values. Fall angles were significantly higher on the 3 µm surface compared to the smooth surface, while single pad adhesive stresses were significantly increased for the 0.3, 3 and 6 µm resin surfaces. Such increases replicate the effects of fine rough surfaces on friction, and probably have a similar explanation. As adhesion has been experimentally shown to scale with pad area [13], and real contact area is increased on a rough surface, such fine rough surfaces could explain an increase in adhesive force. The increase in adhesion on the PDMS surfaces with higher densities of asperities probably has a similar explanation. On the surfaces with larger particle sizes, both fall angles and the adhesive stresses of single pads are reduced. These reductions may be due to loss of close contact and the presence of gaps in the fluid layer (seen with larger beads in the IRM experiments). There are two possible explanations for the presence of air bubbles under the pad in this situation, which are not mutually exclusive. First, the frogs may not be able to produce enough fluid to fill the increased space around the asperities. Second, the increased roughness may increase the drainage of fluid from the pad. Whatever the explanation, wetting the rough surface resulted in a significant increase in adhesion (Figure 7) as it would have reduced the numbers of air bubbles trapped below the pad. Reductions in the fall angles may also be caused by reductions in the friction forces. As described above, this would lead to sliding, which would cause an increase in the pad/surface angle with a concomitant reduction in the pull-off force (peeling theory of Kendall [34]). Interestingly, on the largest asperities tested, (the 562.5 µm beads surface), the frogs began to show an increase in adhesive ability (also noted by Barnes et al. [15]). This could be due to the beads’ diameter being close to that of the frog’s toe pads (area ca 4 mm^2^); therefore close contact could be made by a significant proportion of the pad on the bead. This effect has also been seen in geckos, where adhesive forces on larger roughnesses were high, due to a restoration of spatula contact on each asperity [35].

### Estimation of the elastic modulus of the toe pad

As described above, friction (and to a lesser extent adhesion) can be enhanced on rough surfaces by interlocking when the size and distribution of asperities matches the pattern of micro- or nanostructures on the surface of the toe pads. Since the toe pad epithelium is made of a relatively soft material, there is the additional possibility that it will conform around asperities, further increasing friction and adhesion. Here we have made direct measurements of the conformation of the toe pad surface to asperities of known size, which can lead to estimates of the Young’s modulus of tree frog toe pads. A similar analysis was used by Lorenz et al. [35], who studied the influence of contamination particles on the adhesion of viscoelastic materials.

Using interference reflection microscopy, which allows one to estimate fluid depth under the pad, it was possible to measure *d,* the distance between the centre of the glass bead and the nearest point of close contact of the pad with the glass surface. This is shown diagrammatically in Figure 6, which also shows some data points that are calculations of the fluid depth from interference minima seen under the microscope. Although the pad would not be expected to exactly follow a sine wave (as drawn on the figure), the data points indicate that the shape of the pad surface approximates to such a curve. The distance *d* is plotted against bead diameter in Figure 5. For small beads, the space surrounding the pad was entirely fluid-filled (plotted in blue). For larger beads, there were frequently air pockets as well (plotted in red).

**Figure 9:**
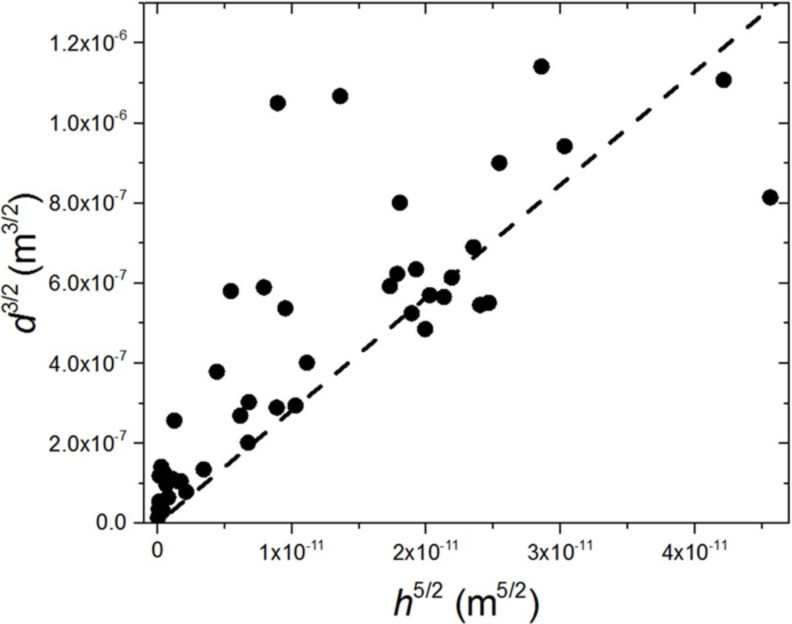
Graph of *d*^3/2^ against *h*^5/2^, where *d* is the distance from the nearest area of close contact to the bead centre and *h* is the diameter of the bead (see Figure 6A). The slope of the line of best fit enables the Young’s Modulus of the pad to be estimated.

To estimate the modulus, first consider the flat toe pad in contact with a flat underlying surface. The introduction of an asperity (e.g., a spherical bead) underneath the tree frog’s foot will lead to an elastic energy penalty associated with the deformation of the toe pad as well as a change in surface energy associated with the interface opening. Assuming the bead and the underlying surface are infinitely stiff compared to the toe pad, only the soft toe pad will deform to accommodate the asperity. The total energy (*U*) of the system is then given by the elastic energy of the toe pad deformation and the surface energy of the opening by *U*_total_ = *U*_elastic_ + *U*_surface_. We approximate the elastic energy according to Hertz theory with an indentation depth of *h* (i.e., bead diameter) and bead radius *R*, leading to *U*_elastic_ = 4/3E*R^1/2^*h*^5/2^. Since the glass bead is taken to be infinitely stiff, here 1/E* = (1 – ν^2^)/*E* where ν is Poisson’s ratio of the toe pad (taken to be 0.5), the surface term is given by the circular opening that is produced by the asperity as *U*_surface_ = π*d*^2^*W*, where *d* is the distance from the bead to the pad in contact (i.e., the circular opening radius) and *W* is the work of adhesion. As the bead size increases, the opening size also increases as suggested by profiles presented in Figure 6. We thus make the assumption that *R* ~ *d*/2. This allows for the minimization of the energy *dU*_total_/*dd*, leading to an approximated relationship between the bead size and opening size of 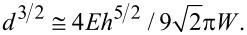 An elastic modulus can be estimated by plotting *d*^3/2^ vs *h*^5/2^ (Figure 9) with the slope scaling with the inverse of the so-called elastocapillary or elastoadhesive length scale, *E*/*W*.

Using a value for the work of adhesion of 70 mN·m^−1^, estimated for tree frog toe pads by Barnes et al. [19], and the slope of the best fit line of the *d*^3/2^ vs *h*^5/2^ plot in Figure 9, the elastic modulus is calculated to be *E* ~ 20 kPa. Indeed, the estimate here is comparable to the results of indentation experiments carried out on tree frogs by Barnes et al. [19] and Barnes et al. [18] which showed equivalently low toe pad elastic modulus values (in the 5–40 kPa range).

Although our results are consistent with prior reports, we note that there are a few points regarding our current analysis that should be taken with precaution. Our assumption of 2*R* ~ *d* excludes additional pre-factors and neglects the interface between the top of the bead and the toe pad that may also influence the calculated modulus. However, by observation of the relationship between *R* and *d* presented in Figure 5, we believe that our assumption is reasonable within a factor of 2. Additionally, the assumed work of adhesion may also be an overestimation since the fluid underneath the toe pad likely changes the interfacial energy. Such values, however, would also be within a factor of about 2. For example, a commonly used value for the work of adhesion of living tissue is 30 mN·m^−1^. If such a value were substituted for the 70 mN·m^−1^ value used above, the resulting modulus would be closer to *E* ~ 10 kPa. Therefore, the elastic modulus found here is within the range of reported values in the literature [18–19,24].

### Comparing performance with other climbing organisms

Several previous studies have examined the effect of surface roughness on the climbing capabilities of other adhesive pad bearing organisms. These include animals with hairy rather than smooth adhesive pads, as well as animals which possess claws as well as adhesive pads. Additionally, there are studies of plant surfaces that have evolved to be anti-adhesive as far as insects are concerned.

The effects of surface roughness on animals with hairy pads (geckos, spiders, insects such as beetles) are reasonably predictable. When the particle size is big enough for the tip of the hair (often a spatula) to make full contact, adhesion and friction forces are similar to those on smooth surfaces. Only when the spatula size is larger than the particle size do the forces decline. For instance, the setal hairs of geckos are built so that they can adapt (acting like a soft material) and conform to rough surfaces of different length scales [36]. Their setae work least effectively on surfaces where the contact area of individual spatulae is split between several asperities (100 to 300 nm root mean squared roughness) [36]. Traction experiments in spiders yielded a similar result, with their adhesive hairs performing poorly when asperity sizes were between 300 nm and 1 µm [37].

The attachment of smooth adhesive pads is more complex. From our data, there is evidence of enhanced adhesion and friction when the wavelength of the surface is similar to that of the pad epithelial cells. Under such conditions, interlocking can occur, as has also been recorded in the euplantulae of stick insects, which consist of frictional ridges [38]. Larger scale roughnesses, on the other hand, appear to result in lower forces. In part this is due to insufficient fluid to fill the gaps between asperities. Kovalev et al. [39] found a similar result in flies (which have hairy pads), where fluid loss was related to the density of asperities. Attachment is also affected by the pad’s stiffness, a low elastic modulus leading to improved moulding of the pad to asperities. The most relevant study in this regard is that of Zhou et al. [17], who tested both the smooth insect pads of cockroaches and the hairy adhesive pads of beetles on nanofabricated surfaces with controlled roughness parameters (the height and spacing of asperities), and found that both parameters affected whether the pads made full or only partial contact with the surface. Such a result indicates that the stiffness of the adhesive pad is another critical parameter in adhesion to rough surfaces, whether the pads are smooth or hairy. This analysis enabled the pad’s effective elastic modulus to be estimated, by a rather more precise methodology than used here. The low values obtained in this study, which are similar to those of other studies [18–19,24], indicate that moulding of the pad surface to asperities will also occur in tree frogs, and will be a major factor in their ability to adhere to rough surfaces. Where insufficient bending occurs (see Figure 8C), air bubbles are likely to be formed, with a consequent reduction in adhesion.

Many climbing organisms utilise claws to climb on rough surfaces, which can interlock with asperities on vertical surfaces – this is seen in geckos [40], spiders [41] and many insects [42–43]. However the effectiveness of a claw is usually dependent on the asperity size being larger than the claw tip diameter [44]. When the claws fail to interlock on the surface, staying attached relies on the adhesive pads of the organism [45]. On the basis of tests with an artificial insect leg, Song et al. [46] claim that, in situations where both claws and pads are both operating, the total force may even exceed the sum of the forces that either system, acting on its own, would have produced.

A number of plants have evolved structures that deter insects (e.g., *Macaranga* trees [12]) or attempt to capture them (e.g., pitcher plants [47]). In both cases, the surfaces will be slippery or otherwise non-adhesive. In many cases, the slipperiness is produced by surfaces covered by epicuticular wax crystals, which break off, contaminating the insect’s adhesive pads [48]. The fine cuticular folds on many surfaces of carnivorous plants may serve a similar function [9].

## Conclusion

In this study, it was shown that tree frog adhesion and friction are significantly affected by surface roughness. Small scale roughness may increase adhesion/friction due to interlocking of asperities with the channels that separate the toe pad epithelial cells. However, in spite of the pad’s low elastic modulus, large scale roughness usually has the opposite effect due to the pad’s inability to mould to the asperities, leading to air bubbles appearing beneath the pad surface. Despite these limitations, tree frogs are still able to generate large forces when landing on a horizontal wooden rod with just one or two toe pads following a jump [49]. They can also climb the narrow twigs and branches of their natural environment by combining adhesion/friction with the ability to grasp even very small twigs [50]. We are thus building up a good understanding of both the underlying mechanisms and the ecology of tree frog adhesive mechanisms. But this study goes further: comparable to the drag reduction mechanisms of snake skin [51], the superhydrophobicity and self-cleaning mechanisms of lotus leaves [52], and the adhesive setae of geckos [53] the toe pads of tree frogs exhibit significant biomimetic potential to advance the technology of surface engineering. This is because they combine high friction under wet conditions [3] with self-cleaning [33]. Their main applications will likely be in the medical field, as Chen et al. already demonstrated their potential for use as surgical graspers [54].

## Experimental

### Experimental animals

Tree frogs *Litoria caerulea* (*n* = 8), were used in these investigations. Their mass was 16.7 ± 6.5 g (mean ± standard deviation) and snout-vent length 57.6 ± 5.5 mm. The frogs were kept in vivaria (30 × 45 × 76 cm high) containing plants and dishes of chlorine-free fresh water at a temperature of approx. 28 °C. They were fed live crickets three times a week, dusted with a multi-vitamin supplement (Nutrobal, purchased from Peregrine Live Foods, Ongar, Essex, England). Before experimentation, the frogs were rinsed in chlorine-free water to remove any dirt or loose dead skin, and carefully blotted dry to prevent the excess water from affecting the frogs’ performance.

### Rough surfaces

Two different kinds of rough surfaces were used in this study, which displayed different topographic features (random asperities and regular patterns).

For whole animal tilting experiments, the rough surfaces (35 × 21 cm) used consisted of eight different grades of polishing discs and sandpaper (made from Aluminium oxide) from multiple sources (3M, USA; Norton abrasives, France; Ultratec, USA), a surface made from a monolayer of 1125 µm glass beads (Ballotini beads, Jencons, VWR International, Leicestershire, UK) and a control surface consisting of a glass plate. Thus, whilst the middle eight of these surfaces had identical surface energies (untested here), the roughest surface differed in both chemistry and the nature of the roughness, since glass beads are spherical, whilst the particles on sandpaper are, by their very nature, much more angular with sharper peaks formed by the particulate asperities on them.

For individual toe pad force measurements, replicas were made of the original surfaces using a low viscosity resin (TAAB laboratories equipment Ltd, UK). The use of resin provided a hard, transparent material that mimicked the sandpapers’ structure by accurately conforming before setting. Their transparency allowed us to measure pad contact area optically. *R*_a_ values for both the originals and the replicas were measured using a Dektak stylus surface profiler (Veeco Dektak 6M Height Profiler, USA. Vertical resolution 0.1 nm) and shown in Table 1.

**Table 1:** List of average roughness values (*R*_a_, in µm) for surfaces used in experiments (polishing discs/sandpaper and their resin copies). The wavelength (width of the asperities) was measured by viewing the surfaces under a microscope.

Surface (approx. asperity height)	Original surface *R*_a_ (µm)	Resin surface *R*_a_ (µm)	Wavelength (µm)

glass cover slip	0.01	0.02	–
0.3 µm	0.21	0.33	1.2
3 µm	1.4	1.6	8.3
6 µm	3.7	2.9	16
16 µm	4.6	5.4	29
30 µm	6.8	6.6	57
58.5 µm	15.5	14.1	100
100 µm	21.5	22	250
425 µm	33.3	–	833.3
562.5 µm (beaded surface)	127^a^	–	1125

^a^The *R*_a_ value of the 562.5 µm (beaded surface) was calculated using the formula in Supporting Information File 1.

**Figure 10:**
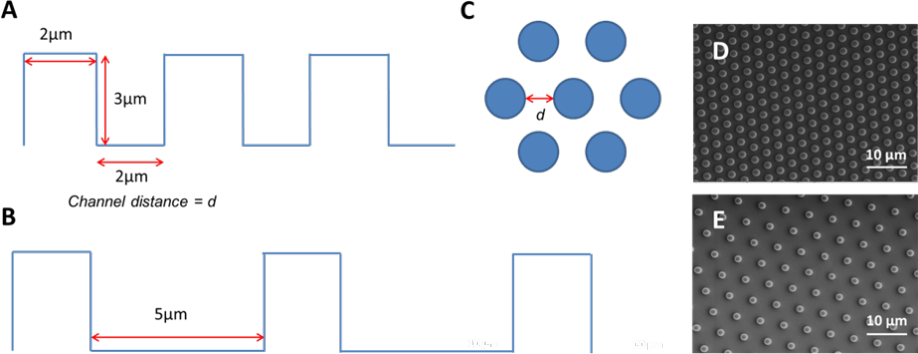
Diagram illustrating the topography of the PDMS structured surfaces. Each pillar on the surface has the same height and diameter, but varies in the gap between each pillar (d). Images A and B display gaps of 2 and 5 µm respectively, while image C shows the layout of pillars from above. Images D and E (gaps of 2 and 5 µm respectively) are SEM images of examples of the PDMS structured surfaces.

Fabricated rough surfaces with regular patterns of asperities were used exclusively for single toe pad force measurements, due to the fact that the surfaces could only be reproduced as small surfaces (ca 20 × 20 mm). These surfaces, made of polydimethylsiloxane (PDMS) were designed to provide transparent surfaces that would allow contact area to be visible through them as well as to provide standardised topographies whose specific dimensions were under experimenter control. The PDMS surfaces were fabricated using moulds kindly made by Dirk Drotlef at the Max Planck Institute for Polymer Research in Mainz. Moulds were created from thin silicon wafers, with micro-patterns etched onto the surface using microlithographic processing. This involves laying down a layer of SU-8 photoresist, then applying a mask to remove specific areas of resistance, and then etching the exposed areas to give the desired patterns. The moulds were negatives of the PDMS patterned surfaces, which produced surfaces consisting of round dimples having fixed measurements for both height (3 µm) and diameter (2 µm) for all surfaces used. Variation between surfaces came in the gap between each asperity. Gap sizes tested were 2, 5, 10 and 30 µm apart, with a smooth PDMS surface acting as a control. SEM imaging confirmed that the surfaces were successfully made (Figure 10D,E). Gap width was selected as the experimental variable, as this was considered the simplest single parameter to change to view the effects on real contact area and thus adhesive force.

PDMS is hydrophobic by nature, and so to cancel out any possible effect that surface energy may have on adhesive forces, the surfaces were plasma treated to make them uniformly hydrophilic before being used each time. Although the precise pad fluid composition is unknown, the capillary forces they use rely on low contact angles with the surface, and so a hydrophobic surface has the potential to be detrimental to adhesion if the fluid is water based, which is still being investigated (as mentioned in the discussion). Plasma treatment involves placing the PDMS sample into a plasma cleaner (Harrick Plasma Inc., NY, USA). Tests were carried out within an hour of plasma treatment.

### Tilting platform apparatus

To test the climbing performance of tree frogs on varying rough surfaces, attachment ability was measured using a tilting platform as used in several previous studies [8,13,16,31]. In this study, the frogs were placed on the rotating platform in a head up posture, and were tilted from a horizontal position (0°) to upside down (180°), to see at what angles the frogs would slip and fall from the board. Rotation was controlled by a Stuart SB3 rotator (Bibby Scientific Ltd, UK), which kept a constant rotation speed of approximately 4 ± 1° s^−1^. The platform itself consisted of a wooden board (21 × 35 cm), to which the different rough surfaces could be attached using binder clips. A smooth glass surface acted as a control. The slip and fall angles for frogs on all of the surfaces were then compared to the control performance. Surface testing was continuously randomised to reduce any potential effect that fatigue could have on results.

The frog was encouraged to stick to the platform to the best of its abilities – a hand being waved around the frog to discourage it from jumping off the board. Whilst the frog was rotated, two angles were measured: when the frog began to slip (indicating maximum friction angle) and when the frog detached from the surface (indicating maximum adhesive angle). The angles were measured using a potentiometer attached to the back of the axial rod of the rotating board, and recorded using a custom LabView interface (LabVIEW Inc., National Instruments, USA). Angles can be converted to forces, so long as the mass of the frog is known, by simple equations (see [13]). These simple experiments have limitations, for the maximum friction force occurs at 90°, and the maximum adhesive force at 180°. Thus maximum friction force for any frog can only be calculated if slipping occurred before 90° and maximum adhesive force if falling occurred before 180°.

### Measuring forces of single pads

Using a similar setup to Crawford et al. [33] the maximum friction and adhesive forces for individual toe pads were measured. A custom built force transducer, composed of strain gauges connected to a bending beam, was used to measure lateral (friction) and normal (adhesive) forces of the toe pad. The plate attached to the bending beam was interchangeable, allowing surfaces of differing roughness to be attached. The resin surfaces could be glued directly onto the bending beam, whilst the PDMS surfaces were attached to a ≈ 1 mm thick piece of flat polyethylene (15 × 15 mm), which had a small opening where the PDMS was situated to avoid impeding the visualisation of contact area. Since the PDMS surface was relatively thick and the hole in the polyethylene small, bending of the PDMS material whilst measuring forces was negligible.

The frog was restrained in a petri dish by a foam cushion which surrounded the body, with one leg extending out from the dish. Light suction on the dorsal side of the toe allowed alignment of the pad with the force plate surface. The frog was then positioned so that the pad rested on the force plate which could be moved relative to it by a pair of computer-controlled precision manipulating stages (model PD-126M, Physik Instrumente, Karlsruhe, Germany). A force feedback system implemented in LabView was programmed to maintain a constant preload (2 mN) for measurements of friction forces. Simple LabView programs (similar to those used in Crawford et al. [33]) were used to move the pad over the force plate so that maximum frictional and adhesive forces could be measured. This involved a proximal lateral drag (5 mm drag at 1 mm s^−1^), followed by a vertical pull off.

Above the setup, a camera (Basler, A602F 100 fps; Ahrensburg, Germany), attached to a stereo microscope (Wild Heerbrugg, Switzerland), allowed the pad area to be visualised during each measurement using coaxial illumination (light travelling through the optical path). This resulted in the pad showing darkly against a bright background. For some samples (particularly the resin surfaces) an additional external source of illumination was required to see the pad contact area with more clarity. Contact area was extracted in conjunction with the force measurements using a customised MATLAB script (Mathworks, Natick, USA) to give force per unit area (stress).

Once a successful trial had been conducted, the frog was either repositioned so that another toe pad was measured, or replaced with another frog. In this way, no pad was tested more than once, avoiding the possibility of reducing the available pad fluid with consequential effects on the force measurements. 30 trials were recorded on each surface.

### Visualising pad contact

In order to gain further understanding of pad contact on rough surfaces, the contact area of the pad around individual asperities was visualised using interference reflection microscopy (IRM). Used on frogs previously by Federle et al. [3], IRM allows one to measure the thickness of the fluid layer beneath the pad and, in our experiments, the extent to which the pad epithelium moulds itself around asperities. The dark (minimum) and light (maximum) interference fringes represent interference between light reflected from the top surface of the coverslip on which the toe pad is resting and from the surface of the toe pad. By comparing images of the same group of cells using two different illuminating wavelengths, 436 nm (blue light) and 546 nm (green light) are most commonly used, it has been shown that the dark centres of each toe pad epithelial cell (Figure 4) are zero order dark fringes, representing distances of at most a few nanometers [3]. The distance between fringes depends on the refractive index of the coverslip and the wavelength of the monochromatic light (λ). The distance (*d*) between individual fringes can be calculated as (adapted from [45]):


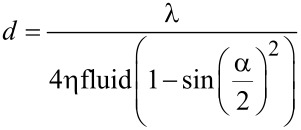


where λ is the wavelength of the light and ηfluid is the refractive index of the toe pad fluid (1.335 according to measurements by [3]), and α = arcsin(INA/ηfluid) with INA being the illuminating numerical aperture.

Using this technique, it was possible to measure the degree of conformation of the soft pad surface to spheres of different sizes, which provided important insights into effects of different roughness scales on adhesion and friction. It also enabled us to estimate the Young’s Modulus of Elasticity for the toe pad epithelium.

Individual frogs were restrained as in the single toe pad force measuring setup in such a way that a single toe pad made contact with a large coverslip (20 × 40 mm). The microscope, a Zeiss Axiovert 200M inverted microscope (Zeiss, Oberkochen, Germany) was set up for IRM, with bandpass filters within the illumination path to provide monochromatic light (546 nm, green light) and a custom built pinhole slider which defined the illumination numerical aperture (measured as 1.001) and reduces stray light. A high power objective lens (×63) was used so that individual cells could be visualised. A camera attached to the microscope (Evolution EX1, Princeton instruments, New Jersey, USA. Image dimensions: 1390 × 1040 pixels) recorded images of pad contact at the cellular level, allowing pad conformity around an asperity to be calculated as described above.

For the experiments, the glass surface was randomly covered with glass beads (Ballotini beads, Jencons, VWR International, UK) of various sizes, ranging from 4.48 µm to 130.51 µm in diameter. The pad was then brought into contact with the surface and the beads to see how well it could conform to the beads present. Using Matlab scripts written specifically for this technique, the distance between the point where the pad is in close contact (seen as the dark patch in the centre of each cell) and the centre of the bead could be measured (Figure 4). This allowed the effect of bead size on the gap size to be investigated. The spacing of the interference fringes from the point of closest contact allows the angle of the pad to the glass coverslip to be calculated, providing more detailed information on the effect of asperities on pad/substrate contact. With large beads, there was insufficient fluid underneath the pad to completely fill the gap, and air bubbles could be seen. Such bubbles would be expected to reduce the adhesive forces that the pads could produce on such surfaces.

### Statistical analysis

Statistical analyses for all the experiments were done using the statistic toolbox in Matlab r2011a. A Lilliefors test was used to determine the normality of each set. Depending on normality of the data, either a student *t*-test or a Wilcoxon rank sum test (also known as a Mann–Whitney U test), was used to compare pairs of data sets, both for the whole animal and single toe pad experiments. For the IRM experiments, linear rank correlation tests were conducted. Data used for multiple tests are corrected using a Bonferroni correction. Ranges of values are indicated by mean ± standard deviation. Boxes in the boxplot figures denote 25th and 75th percentiles, the whiskers display 99% of the data, the middle line shows the median, while outliers are shown as plusses (+).

## Supporting Information

Supporting information explains the calculations of the average roughness (*R*_a_) of a uniform monolayer of beads on a surface.

File 1 Calculating the *R*_a_ of a uniform bead monolayer surface.
