# Supplementary material for: When the going gets rough – studying the effect of surface roughness on the adhesive abilities of tree frogs
Source: Beilstein J Nanotechnol. 2016 Dec 30;7:2116–31. doi: 10.3762/bjnano.7.201 (PMC5238669; doi:10.3762/bjnano.7.201)
Supplement: File 1 — Calculating the Ra of a uniform bead monolayer surface. [file Beilstein_J_Nanotechnol-07-2116-s001.pdf]

Supporting Information

for

When the going gets rough – studying the effect of surface roughness on the adhesive abilities of tree frogs

Niall Crawford[1], Thomas Endlein[2], Jonathan T. Pham[3], Mathis Riehle[1] and W. Jon P. Barnes*[1]

Address: [1]Centre for Cell Engineering, Institute of Molecular Cell and Systems Biology, University of Glasgow, Glasgow, Scotland, UK, [2]Max Planck Institute for Intelligent Systems, Stuttgart, Germany and [3]Max Planck Institute for Polymer Research, Mainz, Germany

Email: Jon Barnes – Jon.Barnes@glasgow.ac.uk

* Corresponding author

Calculating the R_a of a uniform bead monolayer surface

In order to calculate the average roughness (R_a) of a surface covered with a monolayer of beads, a formula was required to calculate the height of the surface throughout a specified area of the surface. The surface used for testing was made

from gluing Ballotini glass spheres (Jencons, VWR International, Leicestershire, UK), with the average diameter of 1125 µm, to a board. The beads were arranged as a tightly-packed monolayer on the surface; this gave the beads a hexagonal configuration, with each bead surrounded by 6 adjacent beads (Figure S1). This configuration leaves small areas between each of the beads where another bead is not present. The adhesive used to keep the beads in place is a relatively thick layer, which has partially filled the gaps between beads. Given this, the size of asperity is recorded as the radius (*r*), with the highest points being the top of the beads (562.5 µm), and the lowest points are at the edges where the beads touch or in the gaps between the beads (measured as 0 µm).

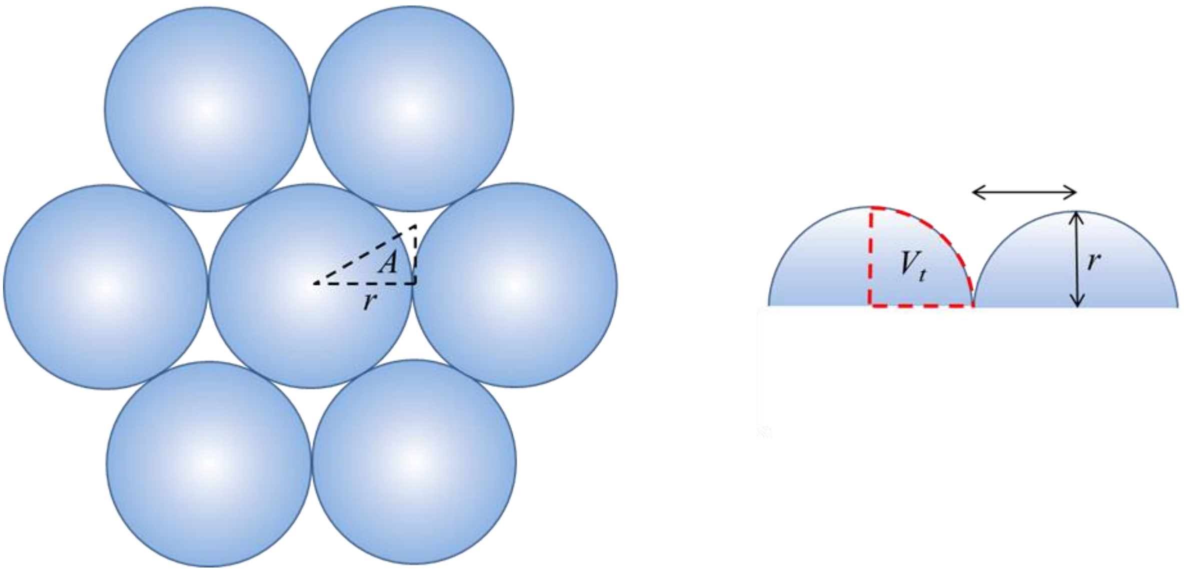

Figure S1: Diagram of the configuration of the monolayer of beads on the surface. The area of interest (*A*) is 1/12 of a hemisphere, which incorporates the radius (*r*) and the volume within the bead (V_t).

The volume of a bead can be calculated using the following equation:

$$V = \frac{4}{3}\pi r_0^3 \quad (1)$$

As we assume the beads to be tightly packed and for the gaps to be filled with the glue, the volume for a hemisphere is more appropriate:

$$V_{hemi} = \frac{2}{3}\pi r_0^3 \qquad (2)$$

To simplify down the calculation, the area of interest can be decreased to 1/12 of each hemisphere; given as:

$$V_t = \frac{1}{18}\pi r_0^3 \qquad (3)$$

From above, the area of interest is represented by the triangle *A* in the figure. This triangle can be mirrored round each bead and throughout the entire area so that the entire surface is correctly measured. The area of the triangle that the 1/12 of the sphere sits on can be calculated as:

$$A = \frac{r_0^2}{2\sqrt{3}} \qquad (4)$$

The average height of a surface made up of hemispheres can be calculated by measuring the average heights over the given area *A*, which is calculated using the following equation:

$$h = \frac{V_t}{A} = \frac{\sqrt{3}}{18}\pi r_0 = \frac{\pi}{3\sqrt{3}} r_0 = 0.605 r_0 \qquad (5)$$

The R_a value of a surface is the mean modulus of the derivation from the average height, the area where the heights have been measured can be divided into positive and negative deviations. For the area tested, the calculated heights can be split into three regions: Above the mean height on the sphere surface, below the mean height on the sphere and area between the beads. These three can be calculated and then brought together to calculate the R_a.

Derivations from the mean height are positive for a sector where $z > h$. This happens for $r < r_1 = \sqrt{1 - h^2} r_0$. The volume of this region is 1/12 of a spherical cap with the radius r_1:

$$V = \frac{\pi h^2}{3}(3r_0 - h) \tag{6}$$

For the volume of the area of bead we are measuring, i.e., 1/12 of the spherical cap:

$$V_1 = \frac{\pi h^2}{36}(3r_0 - h) \tag{7}$$

The second volume is the region of the sphere outside of the spherical cap r_1, and this can be determined via subtraction of the spherical cap and the volume directly beneath it:

$$V_2 = V_{ring} - (V_t - V_1 - V_1') \tag{8}$$

Where V_1' is the volume under h for $r < r_1$:

$$V_1' = \frac{\pi}{12} r_1^2$$

(9)

$$V_{ring} = \frac{\pi}{12}(r_0^2 - r_1^2)h$$

(10)

The final calculation is for the region outside of the sphere, but inside the triangle *A*:

$$V_3 = (\frac{1}{2\sqrt{3}} - \frac{\pi}{12}) r_0^2 h$$

(11)

Finally, to calculate the mean deviation (R_a), all of the volumes are added together and divided by the area of the triangle:

$$R_a = \frac{V_1 + V_2 + V_3}{A}$$

(12)

As you would expect with deviations from a mean, $V_1 - V_2 - V_3 = 0$.
